# Enhancing NUE in Corn Through Optimized Sensor-Based Prescription Maps

**DOI:** 10.3390/s25103148

**Published:** 2025-05-16

**Authors:** Salman Mirzaee, Ali Mirzakhani Nafchi

**Affiliations:** 1 Department of Agronomy, Horticulture and Plant Sciences, College of Agriculture, Food and Environmental Sciences, South Dakota State University, Brookings, SD 57007, USA; 2 Departments of Agricultural & Biosystem Engineering, College of Agriculture, Food and Environmental Sciences, South Dakota State University, Brookings, SD 57007, USA

**Keywords:** flat-rate method, nitrogen environmental risks, remote sensing data, variable-rate method

## Abstract

Enhancing nitrogen use efficiency (NUE) through optimized application methods can benefit agronomic productivity and environmental sustainability. This study examined three nitrogen application strategies, flat rate, soil-based sensing, and remote sensing-based prescription maps, for corn in southeast South Dakota, USA. Soil-based sensing utilized an electrical conductivity (EC) sensor while the normalized difference vegetation index (NDVI) was extracted from remote sensing data using Sentinel-2 images to create different zones. In the flat-rate method, nitrogen is applied uniformly at all plots, regardless of field variations. On the other hand, the sensor-based methods recommended variable rates of nitrogen applications to address field variations. The results of the present study showed that remote sensing-based methods significantly identify field variations as different zones (*p* < 0.05). The remote sensing-based method improved NUE compared to the flat-rate method, with increases of 2.21, 29.24, 29.6, and 82.09% in zones 1, 2, 3, and 4, respectively. However, adjusting the spatial and temporal nitrogen requirement rates using a soil-based sensor was difficult. The findings suggest remote sensing-based method can offer nitrogen optimization by incorporating in-season environmental variability, enhancing agronomic efficiency and sustainability.

## 1. Introduction

The annual global requirement for nitrogen fertilizers is more than 100 million tons, and it has been projected to increase by more than 200 million tons by 2050 to meet global demand [1,2]. Corn (*Zea mays* L.) is one of the most widely grown crops in the world, covering an area of about 190 million ha. It is also an important source of human food, animal feed, and biofuel [3]. Globally, corn is the largest consumer of nitrogen fertilizers, accounting for 16.2% of the total usage [4]. In the United States alone, it consumes almost half of the country’s total nitrogen fertilizer supply [4]. The U.S. still loses nearly half of the applied nitrogen fertilizer due to inefficiencies in its utilization [5,6,7]. These losses cause severe soil degradation, environmental pollution, and ecological damage, such as water pollution and the emission of greenhouse gases. In this regard, Kahrl et al. [8], Billen et al. [9], and Raza et al. [10] concluded that it is essential to adopt advanced nitrogen fertilizer management approaches to improve crop productivity and optimize nitrogen use efficiency and reduce associated environmental risks of nitrogen losses.

Enhancing Nitrogen Use Efficiency (NUE) is a critical strategy for increasing crop productivity while decreasing environmental degradation [11,12,13]. The simplest approach to determine NUE is by calculating the ratio of nitrogen uptake in crop production to nitrogen input [13]. Achieving higher NUE requires nitrogen applications according to the specific needs of plants in the field. The approach of variable-rate nitrogen application has proved effective in enhancing NUE, increasing grain yields, improving crop quality, and maximizing economic returns while reducing nitrogen overload in the environment [13]. Despite these benefits, Koch et al. [14] and Guerrero et al. [15] note that its adoption rate remains relatively low in the United States.

According to McFadden et al. [16] reports, the adoption of variable-rate fertilizer application was 28.2% for corn in 2016, 13.9% for soybean in 2018, 14.6% for winter wheat in 2017, 14.3% for cotton in 2019, and only 8.0% for sorghum in 2019. Thus, most fertilizer applications in the U.S. still depend on flat-rate methods. Flat-rate nitrogen application is a conventional and simple method in which the same quantity of nitrogen fertilizer is uniformly spread across a whole field. This application is easy and widely adopted; however, it usually leads to an excessive amount of nitrogen being applied to ensure yield potential. The overapplication of nitrogen, on the other hand, reduces profitability for farmers [17,18] and is considered to contribute greatly to environmental contamination [19]. Conversely, sub-optimal fertilizer applications in parts of the field lead to lower crop yield [15]. For optimizing nitrogen fertilizer application, there is a need to use precision agriculture techniques [13]. To address these inefficiencies, precision agriculture techniques are essential for optimizing nitrogen fertilizer application.

A fundamental concept in precision agriculture is the creation of management zone maps. The zones are determined areas within a field that have similar agronomic characteristics and respond to inputs. The basic methods are soil-, historical yield-, and remote sensing-based management zones map. The soil-based management zone map is a static method and historical yield- and remote sensing-based can provide dynamic data. In this way, static (soil)-based methods and historical yield-based management zone maps have some significant advantages. For example, they are relatively easy to implement, integrate well with existing precision agriculture technologies, and provide a practical way to account for spatial variability across fields [13,20,21,22]. However, these methods have significant limitations. One major of them is their reliance on past data, which makes it difficult to adjust nitrogen application rates in response to year-to-year variability in weather, crop growth, and other dynamic in-season conditions [20,21,22]. As a result, they may not effectively capture real-time nutrient needs and lead to sub-optimal nitrogen use efficiency and potential environmental impacts [13]. Therefore, it is important to focus on these challenges for optimizing nitrogen application rates. The objectives of this study were to evaluate different nitrogen application methods and determine the optimum method to enhance NUE.

## 2. Material and Methods

### 2.1. Study Area

**Figure 1:**
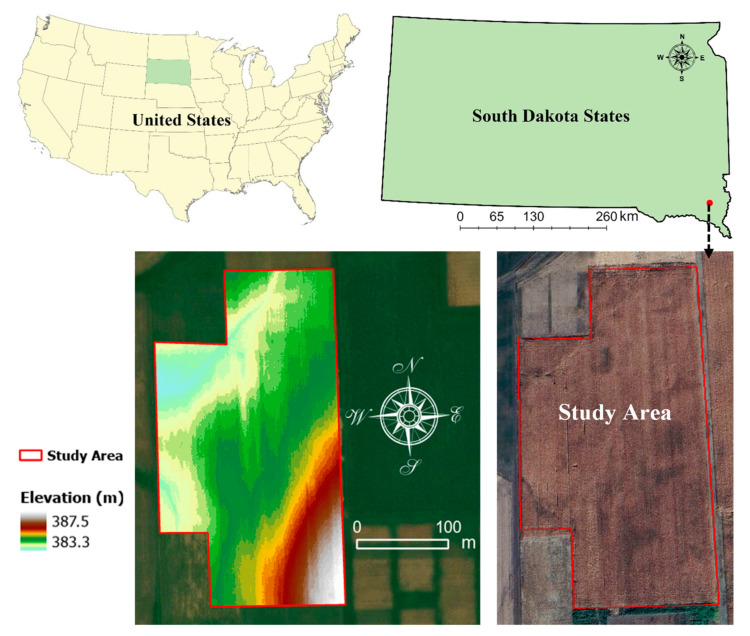
Location of the study area in South Dakota State, USA.

The field experiment was carried out at the southeast research farm of South Dakota State University, the southeast part of South Dakota State, United States. It was carried out in the geographic coordinates of 43°3′8.01″ N latitude and 96°54′3.47″ W longitude (Figure 1). The field covers a total area of 6.3 ha. It is situated within a region characterized by moderate elevation variability, ranging from 383.3 to 387.5 m above sea level. The study area climate supports rainfed corn production with no-till farming.

**Figure 2:**
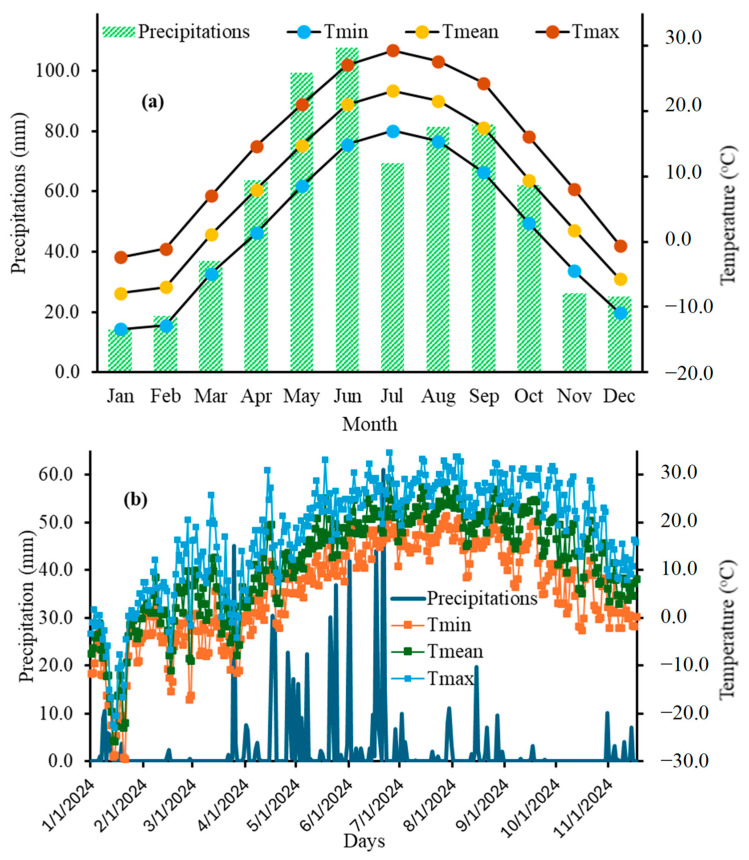
The average monthly precipitation and temperature from 2000 to 2023 (**a**) and the daily precipitation and temperature for 2024 (**b**).

The study area is located in a humid continental climate zone. The region experiences four distinct seasons with significant temperature and precipitation variations throughout the year. The corn-growing season typically varies from May to September. The majority of annual precipitation occurs during the late spring and summer months (Figure 2a). On average (from 2000 to 2023), the total rainfall in the corn-growing season was 439 mm (Figure 2a). Additionally, the monthly average temperature (from 2000 to 2023) varied from 14.79 °C in May to 23.15 °C in July (Figure 2a). Furthermore, the daily rainfall and temperature variations for 2024 (the current season for corn production) for the study area are presented in Figure 2b. Total monthly rainfall in April, May, June, July, August, and September was 149.5, 145.4, 200.0, 40.2, 46.4, and 4.89 mm, respectively (Figure 2b).

### 2.2. Experimental Design

**Figure 3:**
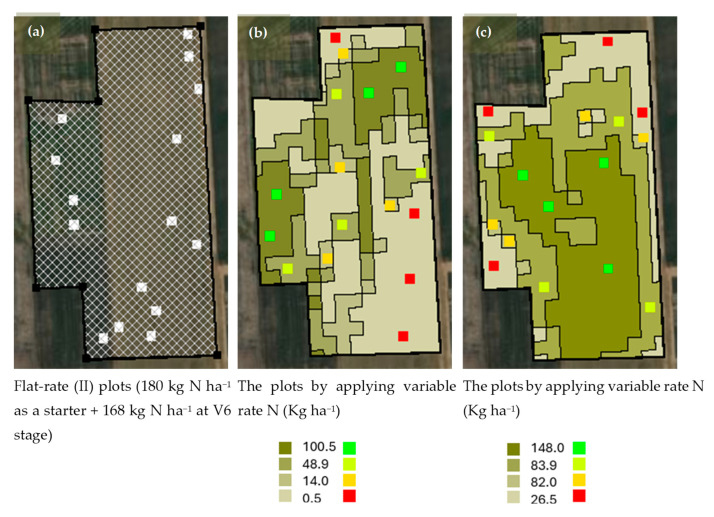
The nitrogen prescription maps created by considering different methods such as (**a**) flat rate, variable rate applying nitrogen using (**b**) soil-based sensor (Electrical Conductivity, EC sensor map), and (**c**) remote sensing-based method (Normalized Difference Vegetation Index, NDVI map).

The experiment started to study nitrogen application methods from May to September 2024. Figure 3 indicates the methods to apply nitrogen fertilizers in the corn field. In the present study, three nitrogen application methods including the flat rate with 180 kg N ha^−1^ as a starter plus 168 kg N ha^−1^ at the V6-8 stage (Figure 3a), the variable rate using EC sensor data (Figure 3b), and the variable rate using NDVI map (Figure 3c).

In the flat-rate method, the yield goal was determined as 250 bu/ac (16.5 t/ha) and then converted to the nitrogen rate using Stanford’s equation and was revised by Lory and Scharf [23] as follows:

(1) $$Nitrogen\;rate\;\left(kg\;{ha}^{-1}\right)=1.121(n\times Yield\;Goal\;(bu\;{ac}^{-1}))$$

**Figure 4:**
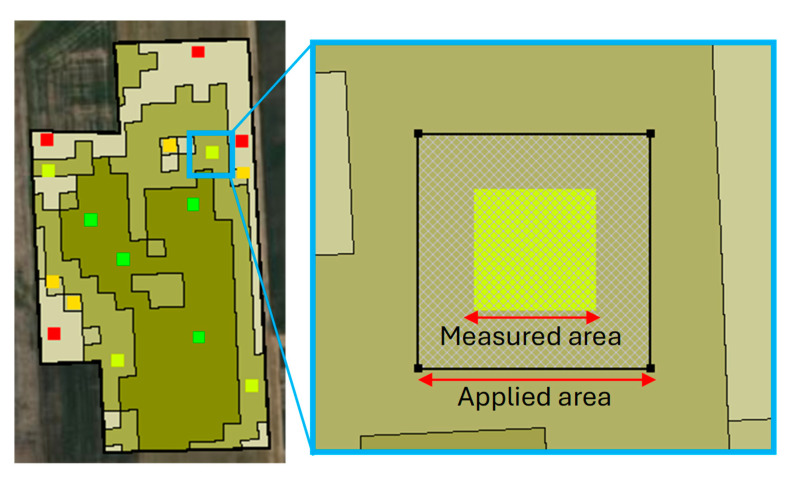
The applied area (20 m) and measured area (10 m) in the present study.

Clark [24] calibrated the *n* coefficient to equal 1.0 for South Dakota State. The final nitrogen rate was determined by considering legume credit, soil test nitrogen, and assuming NUE is equal to 0.6. The nitrogen application rates for both the soil- and remote-sensing-based sensors were determined by analyzing multi-year historical yield data. In the variable-rate application methods, different management zones with a specific nitrogen rate were arranged in a completely randomized design (CRD) with four replications. The nitrogen fertilizer rates for management zones included 0.5, 14.0, 48.9, and 100.5 kg N ha^−1^ in an EC sensor-created nitrogen prescription map (Figure 3b) and 26.5, 82.0, 83.9, and 148.0 kg N ha^−1^ in an NDVI-based-created nitrogen prescription map (Figure 3c). There were 47 plots in total, and each applied and measured plot was 20 m × 20 m and 10 m × 10 m, respectively (Figure 4).

### 2.3. Soil Sampling and Analysis

**Figure 5:**
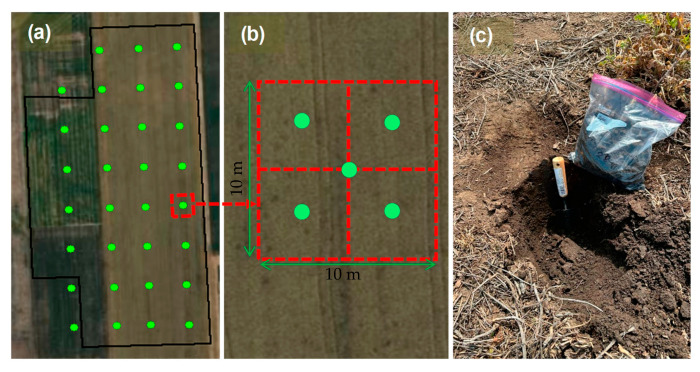
Distribution of soil sampling points at the study area (**a**), zoomed-in schematic of a single sampling plot where soil cores were collected and composited (**b**), and collected soil samples (**c**).

The study field was sampled using a grid sampling method (Figure 5). At each grid point (approximately 0.01 ha), five soil sub-samples were collected from a depth of zero to 30 cm and thoroughly mixed to form a composite sample. In total, 31 soil samples were collected, with an average distance of approximately 45 m between sampling points (Figure 5). The collected soil samples were air-dried, during which visible plant residues, stones, and other impurities were removed. Subsequently, the samples were sieved through a 2 mm mesh to prepare them for analysis. Particle size distribution, including clay, silt, and sand fractions, was determined using the hydrometer method [25]. Soil pH was measured using a 1:1 (Soil:Water) ratio [26]. Soil salinity was analyzed by measuring the electrical conductivity (ECe) of a saturated soil extract at room temperature (25 °C), following standard protocols [27]. Soil organic matter (SOM) content was determined by loss on the ignition method [28]. Additionally, total nitrogen (TN) was measured using the method described by Nelson and Sommers [29]. Available phosphorus (P) was quantified using Olsen’s method [30] and Mehlich-3 extraction procedure [31]. The exchangeable potassium (K) was determined by Jackson’s procedure [32].

### 2.4. Gathering Data by Sensors

**Figure 6:**
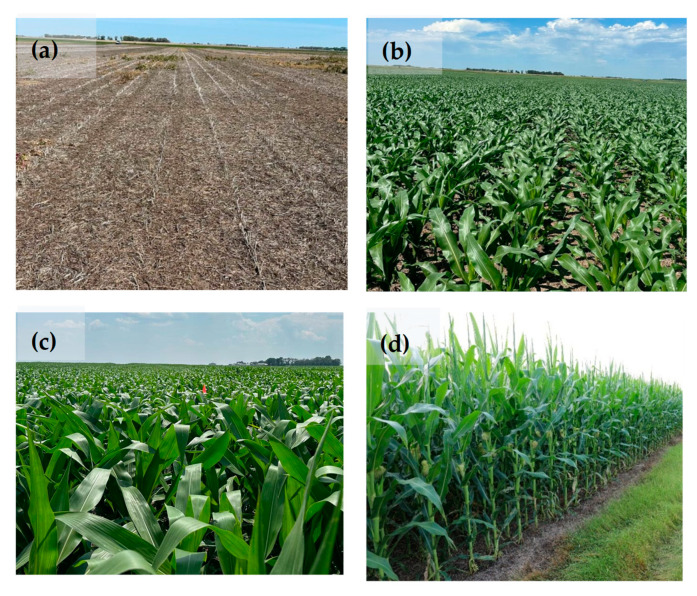
Different corn growth stages such as planting time (**a**), V6 (**b**), V10 (**c**), and reproductive (**d**).

**Figure 7:**
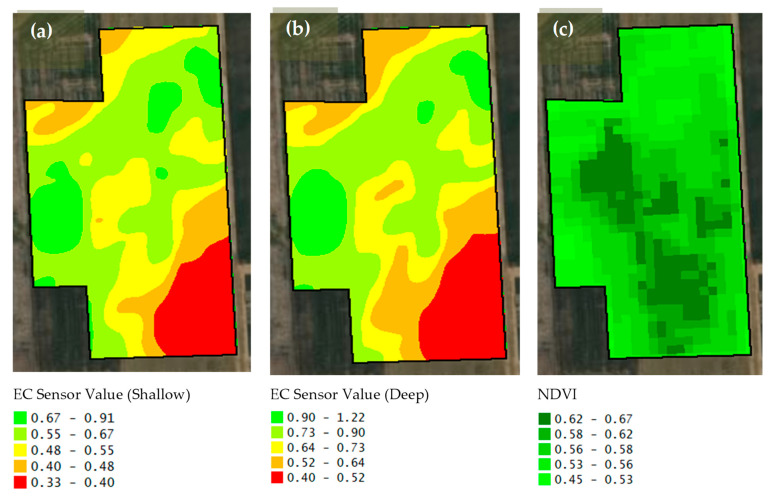
The different types of sensor values come from EC (Electrical Conductivity) sensor value for 0.5 m soil depth (Shallow) (**a**), EC sensor value for 1.0 m soil depth (Deep) (**b**), and NDVI (Sentinel 2 on 10 July 2024) (**c**).

Figure 6 shows some stages of corn growth. First, the present study tried to collect soil type data (Figure 7a). To achieve this, the EM38-MK2, an advanced electromagnetic induction (EMI) sensor is manufactured by Geonics Ltd. in Mississauga, ON, Canada, was used to measure apparent electrical conductivity (ECa) at corn planting time (Figure 7a,b). Second, to monitor temporal variations, Sentinel-2 satellite images were utilized to extract NDVI maps (Figure 7c). The NDVI maps were extracted at key stages of the corn growth cycle, including the V6 to V8 growth stage (Figure 6b) on 10 July 2024, and the reproductive stage (Figure 6d) on 30 July 2024. In general, NDVI values varied between −1.0 and 1.0. The negative values show water surfaces, clouds, snow, etc. The values from 0.10 to 0.15 indicate bare soil. The values above 0.20 show the vegetation [33].

### 2.5. Yield Sampling and Map

The corn yield was measured by sampling from plots and the yield map was extracted from a combined yield monitor system. In the present study, the corn yield was measured in the plots with 10 m in length and 10 m in width as shown in Figure 4.

### 2.6. Nitrogen Use Efficiently (NUE)

The *NUE* in the present study was calculated using the following equation [13]:

(2) $$NUE=\frac{N_{yield}}{N_{input}}$$

where, $N_{yield}$ is the nitrogen uptake by crops and $N_{input}$ is the nitrogen input.

### 2.7. Statistical Analysis

Data analysis was conducted using R v:4.3.2 software using *agricolae 1.3-7* and *stats 4.3.2* packages for functionality on experimental design and statistical analysis. A one-way analysis of variance (ANOVA) was carried out to identify significant differences between methods. Then treatments were tested by using Duncan’s test with a significant level of *p* < 0.05. The effects of nitrogen application methods on the corn yield were comprehensively evaluated.

## 3. Results

### 3.1. Soil Features Results

**Figure 8:**
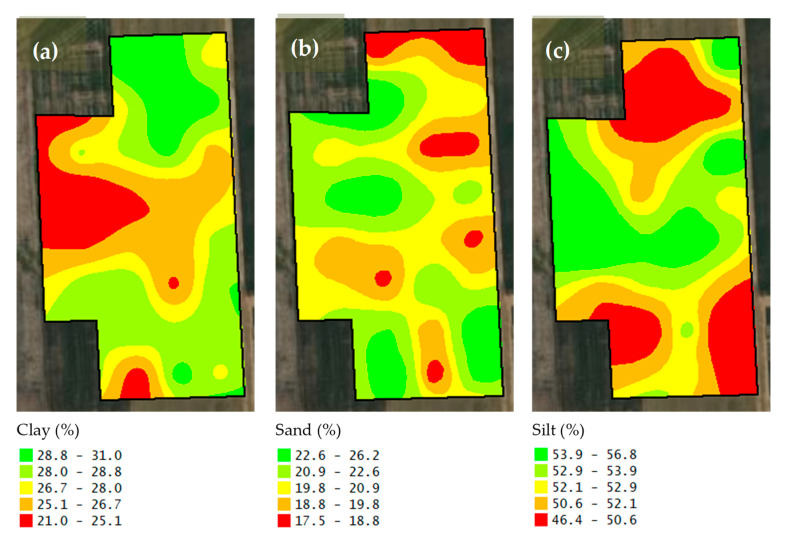
The maps of clay (**a**), sand (**b**), and silt (**c**) contents in the study area.

**Figure 9:**
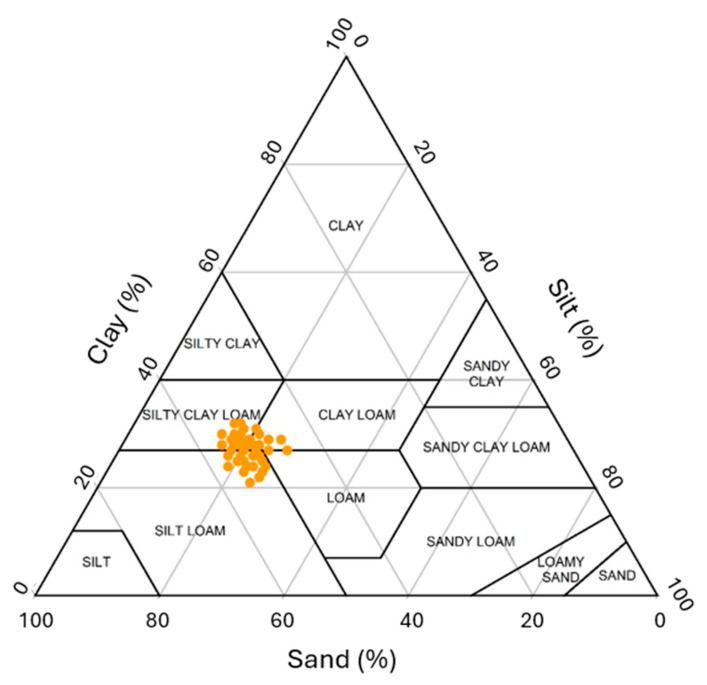
The USDA textural distribution of the soils in the study area.

**Figure 10:**
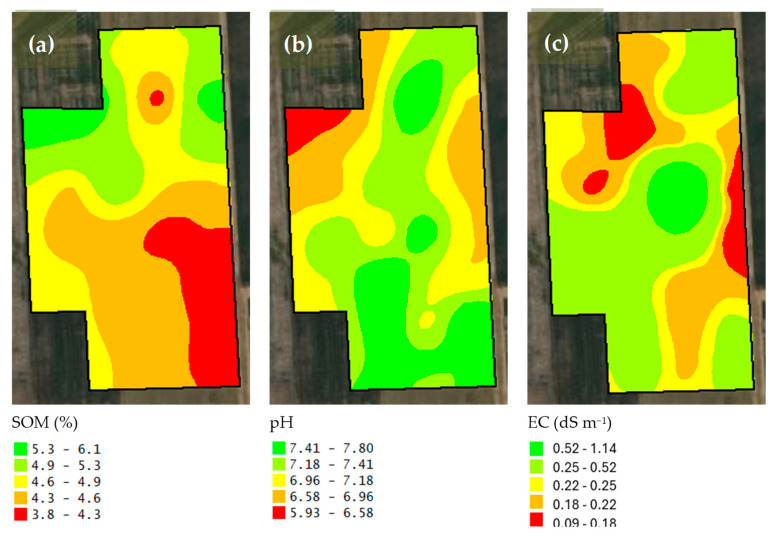
The maps of soil organic matter (**a**), pH (**b**), and electrical conductivity (**c**).

**Figure 11:**
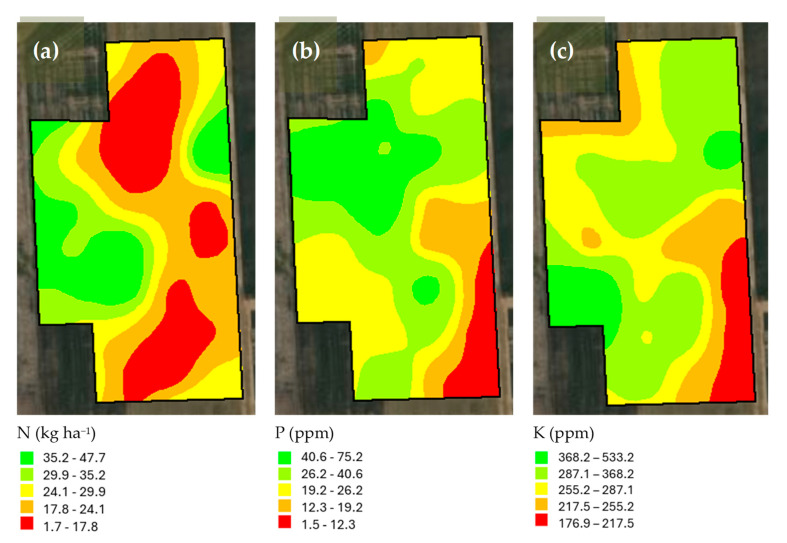
The maps of soil nutrient elements such as nitrogen (**a**), phosphorous (**b**), and potassium (**c**).

Descriptive statistics of the studied soil features are given in Table 1. In addition to the soil particle fractions such as clay, silt, and sand contents in Table 1, the maps of soil physical properties are presented in Figure 8. According to the U.S. Soil Taxonomy classification as included in Figure 9, the soil texture classes of this study were clay loam, silty clay loam, and silt loam. Moreover, the maps of soil organic matter, pH, and electrical conductivity are shown in Figure 10. The standard deviation for soil organic matter, pH, and electrical conductivity were 0.4%, 0.16, and 0.4 dS m^−1^ (Table 1). Furthermore, nutrient elementary maps such as soil nitrogen, phosphorus, and potassium are presented in Figure 11. The mean nitrogen was 25.0 kg ha^−1^, with values ranging from 9.0 and 50.4 kg ha^−1^ (Table 1). From Table 1, it follows that the coefficient of variation (CV) for nitrogen was 47.5 kg ha^−1^. Therefore, soil nitrogen rates showed a broad range of values. It is one of the important characteristics to consider in determining the best method to develop a nitrogen variable-rate prescription map.

### 3.2. Performance of Different Developed Nitrogen Application Methods

#### 3.2.1. Corn Yield Under Different Nitrogen Application Methods

**Figure 12:**
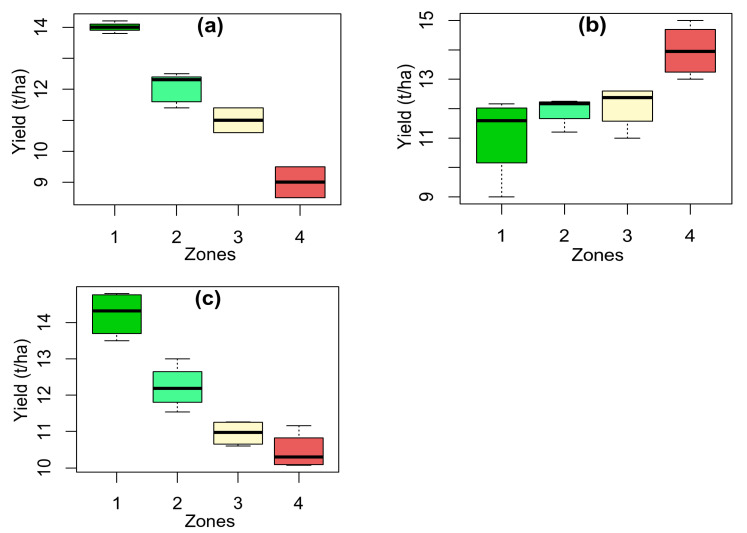
The boxplots of corn yield at different zones using varied nitrogen application methods such as (**a**) flat-rate method, variable-rate application methods using (**b**) soil-based sensors (Electrical Conductivity, EC sensor map), and (**c**) remote sensing-based method (Normalized Difference Vegetation Index, NDVI map).

The ANOVA analysis showed significant differences between the zones at *p* < 0.001, *p* < 0.01, and *p* < 0.001 for flat rate, soil-based sensor method, and remote sensing-based method, respectively. Table 2 and Figure 12 illustrate the results of corn yield under different nitrogen application methods for different zones. Applying nitrogen by flat-rate method resulted in yields ranging from 8.50 to 14.04 t ha^−1^ as shown in Figure 12. As can be seen from Table 2, the corn average yield was significantly (*p* < 0.05) different for zones by this method. Zone 1 achieved the highest yield of 14.04 t ha^−1^, whereas Zone 4 had the lowest yield at 9.50 t ha^−1^ (Figure 12). Similar trends were observed with the remote sensing-based variable-rate method (Table 2 and Figure 12). However, yields in Zones 3 and 4 did not differ significantly. In contrast, the soil-based sensor method (mostly according to the shallow EC sensor map as shown in Figure 7a) was not able to demonstrate corn yield according to the obtained yield (Table 2 and Figure 12). For example, the highest yield was for a low-quality zone and vice versa (Table 2 and Figure 12).

#### 3.2.2. NUE Under Different Nitrogen Application Methods

**Figure 13:**
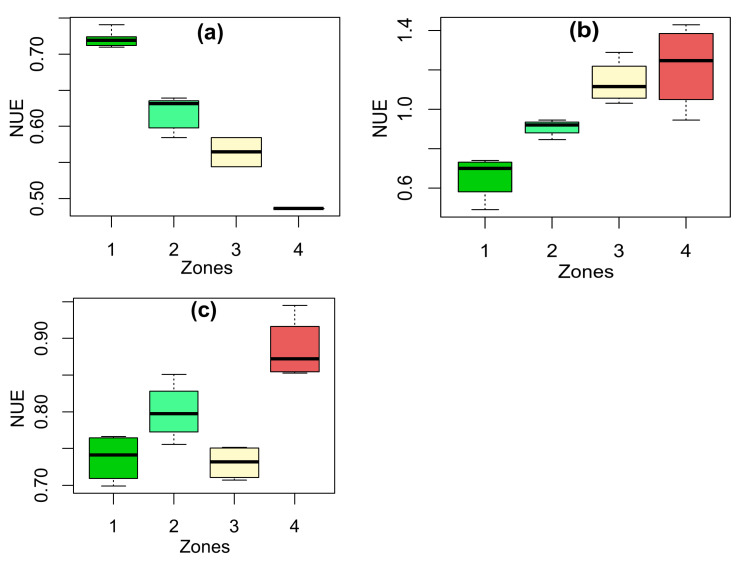
The boxplots of NUE at different zones using various nitrogen application methods such as (**a**) flat-rate method, variable-rate application methods using (**b**) soil-based sensors (Electrical Conductivity, EC sensor map), and (**c**) remote sensing-based method (Normalized Difference Vegetation Index, NDVI map).

Figure 13 and Table 3 show the results of NUE under different zones by applying nitrogen in various methods such as flat rate, soil-based sensors (EC sensor map), and remote sensing-based methods (NDVI map). According to the results shown in Table 3, there is a significant decrease in NUE from Zone 1 to Zone 4 using the flat-rate method (*p* < 0.05). Zone 1 demonstrates higher NUE due to its greater nitrogen uptake capacity linked to its higher yield potential. The analysis indicates that other zones do not achieve nitrogen uptake as effectively as Zone 1, primarily due to their lower inherent yield potentials (Table 3).

In the basis of Table 3, the remote sensing-based method indicates that the corn crops in Zones 4, 2, and 3 or 1 significantly uptake different rates of nitrogen (*p* < 0.05). The highest NUE were for Zone 4 and Zone 3 (Table 3). The reason for the highest NUE in Zone 4 is related to applying the lowest nitrogen because of the lowest yield potential. Generally, this method effectively differentiates NUE in different zones and results in higher efficiency rates compared to the flat-rate method. This enhanced performance is attributed to the remote sensing-based variable-rate method’s ability to consider in-season variations. However, the soil-based sensors method (EC sensor map) does not work with the observed field yield patterns (Table 2 and Table 3). It is due to the inability of this method to take in-season conditions.

### 3.3. Comparison of Flat-Rate- and Remote-Sensing-Based Nitrogen Application Methods

The results of the comparison between flat-rate- and remote-sensing-based nitrogen application methods are presented in Table 4. The results of Table 4 showed that there is no statistically significant difference in corn yield between those nitrogen application methods in Zone 1, 2, and 3. However, the results were different in Zone 4 (Table 4). The reason for lower corn yield in Zone 4 using a flat rate is related to higher available nitrogen rather than the yield potential. However, the performance of nitrogen application methods for NUE was different than corn yield. As can be seen from Table 4, there is a significant difference between those methods except Zone 1. It clearly shows the NUE of Zone 2, 3, and 4 in the remote sensing-based method were significantly higher than the flat-rate method (Table 4). Therefore, the flat-rate method showed a low performance in zones 2, 3, and 4 because of applying the same rate of nitrogen fertilizer for all of the zones with different yield potential.

### 3.4. Analysis of Yield Map

**Figure 14:**
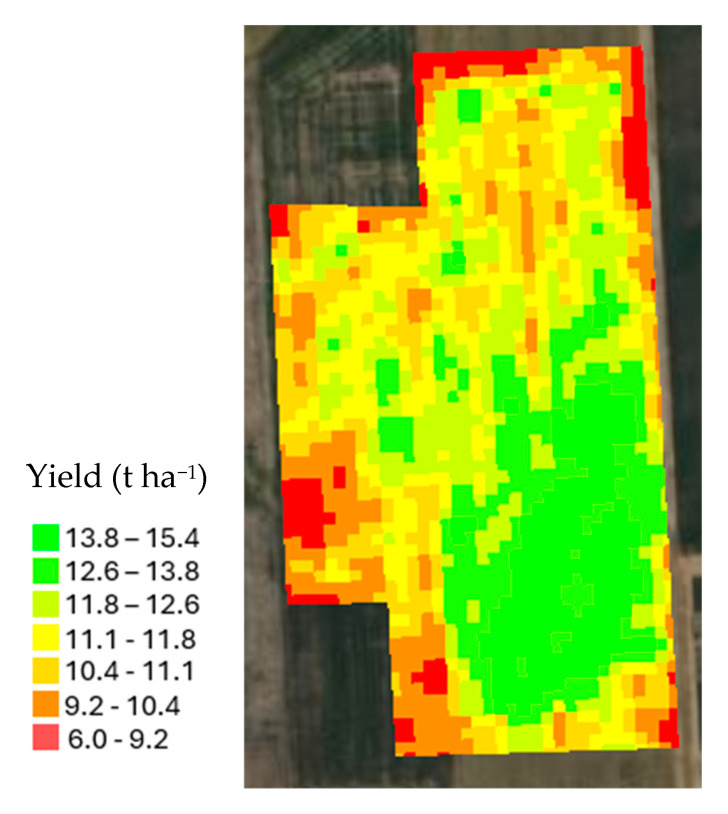
The yield map of the study area.

Figure 14 presents a detailed yield map of the study area, derived from data collected using a yield monitor system. In the southeastern part of the field, the yield peaks significantly, with values ranging from 12.6 to 15.4 t ha^−1^. It shows there were optimal growing conditions in that area. Conversely, lower yield zones are evident in some parts of the northern and southwestern sections of the field, where yields drop to between 6.0 and 10.4 t ha^−1^ (Figure 14). These lower yields could be attributed to less favorable growing conditions. A middle range of yields (between 10.4 and 12.6 t ha^−1^) are mostly in the central and north parts of the study area (Figure 14).

## 4. Discussion

Traditionally, farmers assessed crop conditions through experience. Today, precision agriculture tools, like smart soil and crop sensors, deliver real-time data to optimize yields and environmental impact [34]. In this way, the results of the present study showed the importance of remote sensing-based methods to optimize corn yield and increase NUE (Table 2, Table 3 and Table 4). It was because of considering the in-season conditions like weather patterns, environmental variability, and any interaction by this method. Recent research reported the efficacy of spectral indices as power tools for estimating crop nitrogen status, which is crucial for optimizing fertilizer use and enhancing crop health [7,20,21,35,36,37,38]. The advantages of spectral indices extracted from remote sensing data are particularly valuable due to their flexibility, portability, cost-effectiveness, timelines, and autonomy [39,40,41]. The limitations can be their resolution, weather conditions, data complexity, and interpretation [38,39,40].

**Figure 15:**
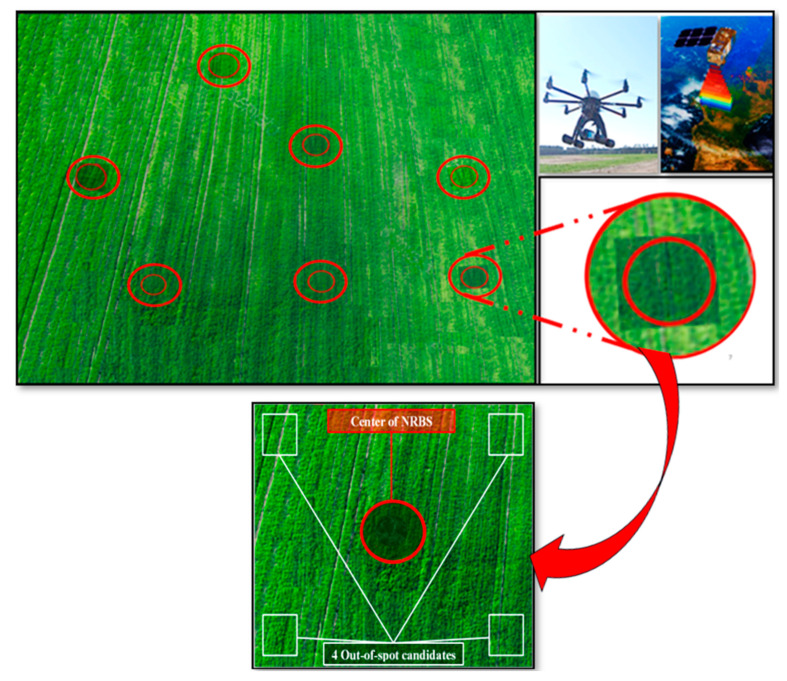
Some Spot Drops Biosensor Nutrient Management in the field [46].

Reviews of Mirzaee and Mirzakhani Nafchi [13] showed that sensor-based nitrogen management systems are generally more environmentally friendly and cost-effective than conventional practices. Compared to traditional methods, using sensor-based nitrogen application methods can improve nitrogen use efficiency by up to 3.7 times [13,42,43]. Smart sensors have also been shown to reduce nitrogen fertilizer use by 10–80% and lower soil residual nitrogen by 30–50%, without compromising wheat grain yield or quality [42]. In this way, Mitra et al. [44] study showed that applying nitrogen using an NDVI sensor with two or three splits indicated higher NUE. NUE increased by more than 15% by considering in-season conditions and optically sensed NDVI [45]. Due to year-to-year yield inversion and variability, soil-based sensors face challenges in adjusting nitrogen applications. Mirzaee and Mirzakhani Nafchi [46] demonstrated that in 57.1% of the field, wet and dry years exhibited inverse yield patterns. In a new method, Spot Drops Biosensor Nutrient Management (SDBNM), nitrogen-rich spots as biosensors integrated with remote sensing data showed strong potential to refine nitrogen recommendations further. SDBNM provides insights into all interactions in real-time nitrogen availability (Figure 15). This methodology captures spatial and temporal variability, making it an effective tool for adaptive nitrogen management. It enhances sustainability and profitability in precision agriculture [46].

## 5. Conclusions

This study tried to evaluate and optimize nitrogen prescription maps for enhancing NUE. For this purpose, flat rate, soil-based sensor (EC sensor map), and remote sensing-based sensor (NDVI map extracted from Sentinel 2 images) were applied. In the present study, results showed that using remote sensing-based method data improved NUE, with increases of 2.21, 29.24, 29.6, and 82.09% in zones 1, 2, 3, and 4, respectively. Enhancing NUE is a key strategy for advancing global food security and promoting environmental stewardship. Ongoing research in precision agriculture technologies, remote sensing, and data-driven decision-making is central to improving nitrogen management practices. Therefore, further studies in diverse environments, cropping systems, and management practices are recommended to evaluate the broader applicability and universality of these results. Continued research in precision agriculture, remote sensing, and data-driven nitrogen management will be vital for building sustainable agricultural systems globally.

## Figures and Tables

**Table 1:** Descriptive statistics for observed soil features.

Soil Features	Min	Max	Mean	Median	Standard Deviation	CV (%) *
Clay (%)	21.0	32.0	27.4	28.0	2.6	9.4
Sand (%)	15.0	27.0	20.4	20.0	2.9	14.2
Silt (%)	46.0	57.0	52.2	52.0	2.4	4.5
SOM (%)	3.8	6.2	4.6	4.5	0.4	9.3
EC (dS m^−1^)	0.13	1.29	0.28	0.26	0.16	58.12
pH	5.9	7.9	7.2	7.2	0.4	5.7
Nitrogen (kg ha^−1^)	9.0	50.4	25.0	22.4	10.6	47.5
Phosphorus (ppm)	3.0	79.0	26.8	22.0	16.2	60.4
Potassium (ppm)	194.0	560.0	283.5	272.0	71.4	25.2

* CV: Coefficient of Variation; EC: Electrical Conductivity; SOM: Soil Organic Matter.

**Table 2:** The corn yields under different nitrogen application methods such as flat-rate and variable-rate application methods.

Nitrogen Application Method	Zone 1	Zone 2	Zone 3	Zone 4
Flat rate	14.04 a	12.08 b	11.00 c	9.00 d
Soil-based sensors (EC sensor map)	11.08 b	11.94 b	12.09 b	13.97 a
Remote sensing-based method (NDVI map)	14.23 a	12.22 b	10.95 c	10.45 c

Different letters in the same row represent a significant level of 0.05.

**Table 3:** The NUE under different nitrogen application methods.

Nitrogen Application Method	Zone 1	Zone 2	Zone 3	Zone 4
Flat rate	0.721 a	0.619 b	0.564 c	0.486 d
Soil-based sensors (EC sensor map)	0.656 c	0.907 c	1.137 a	1.216 b
Remote sensing-based method (NDVI map)	0.737 c	0.800 b	0.731 c	0.885 a

Different letters in the same row represent a significant level of 0.05.

**Table 4:** The corn yield and NUE under different nitrogen application methods.

Parameters	Nitrogen Application Method	Zone 1	Zone 2	Zone 3	Zone 4
Corn Yield	Flat rate	14.04 a	12.08 b	11.00 c	9.00 d
	Remote sensing-based method (NDVI map)	14.23 a	12.22 b	10.95 c	10.45 c
NUE	Flat rate	0.721 c	0.619 d	0.564 e	0.486 f
	Remote sensing-based method (NDVI map)	0.737 c	0.800 b	0.731 c	0.885 a

Different letters in the same row represent a significant level of 0.05.

## Data Availability

Data will be made available on request.
